# A bile-based microRNA signature for differentiating malignant from benign pancreaticobiliary disease

**DOI:** 10.1186/s40164-023-00458-3

**Published:** 2023-12-01

**Authors:** Mireia Mato Prado, Jisce R. Puik, Leandro Castellano, Elena López-Jiménez, Daniel S. K. Liu, Laura L. Meijer, Tessa Y. S. Le Large, Eleanor Rees, Niccola Funel, Shivan Sivakumar, Stephen P. Pereira, Geert Kazemier, Babs M. Zonderhuis, Joris I. Erdmann, Rutger-Jan Swijnenburg, Andrea Frilling, Long R. Jiao, Justin Stebbing, Elisa Giovannetti, Jonathan Krell, Adam E. Frampton

**Affiliations:** 1 Division of Cancer, Department of Surgery & Cancer, Imperial College London, London, UK; 2 UK Dementia Research Institute, Institute of Neurology, University College London, London, UK; 3 Department of Surgery, Amsterdam UMC Location Vrije Universiteit Amsterdam, De Boelelaan 1117, Amsterdam, The Netherlands; 4 Cancer Center Amsterdam, Imaging and Biomarkers, Amsterdam, The Netherlands; 5 School of Life Sciences, University of Sussex, John Maynard Smith Building, Falmer, Brighton, BN1 9QG UK; 6 Department of Translational Research and New Technologies in Medicine and Surgery, University of Pisa, Pisa, Italy; 7 Oncology Department and Institute of Immunology and Immunotherapy, Birmingham Medical School, University of Birmingham, Birmingham, B15 2TT UK; 8 Institute for Liver & Digestive Health, Royal Free Hospital Campus, University College London, London, UK; 9 HPB Surgical Unit, Department of Surgery & Cancer, Imperial College London, London, UK; 10 Department of Biomedical Sciences, Anglia Ruskin University, Cambridge, UK; 11 Cancer Pharmacology Lab, Fondazione Pisana per la Scienza, Pisa, Italy; 12 HPB Surgical Unit, Royal Surrey NHS Foundation Trust, Guildford, Surrey, UK; 13 Section of Oncology, Dept. of Clinical & Experimental Medicine, FHMS, University of Surrey, Guildford, UK

**Keywords:** Cholangiocarcinoma, Pancreatic ductal adenocarcinoma, microRNA, Biomarker, Biliary stricture, Bile

## Abstract

Differentiating between pancreatic ductal adenocarcinoma (PDAC) and cholangiocarcinoma (CCA) is crucial for the appropriate course of treatment, especially with advancements in the role of neoadjuvant chemotherapies for PDAC, compared to CCA. Furthermore, benign pancreaticobiliary diseases can mimic malignant disease, and indeterminate lesions may require repeated investigations to achieve a diagnosis. As bile flows in close proximity to these lesions, we aimed to establish a bile-based microRNA (miRNA) signature to discriminate between malignant and benign pancreaticobiliary diseases. We performed miRNA discovery by global profiling of 800 miRNAs using the NanoString nCounter platform in prospectively collected bile samples from malignant (*n* = 43) and benign (*n* = 14) pancreaticobiliary disease. Differentially expressed miRNAs were validated by RT-qPCR and further assessed in an independent validation cohort of bile from malignant (*n* = 37) and benign (*n* = 38) pancreaticobiliary disease. MiR-148a-3p was identified as a discriminatory marker that effectively distinguished malignant from benign pancreaticobiliary disease in the discovery cohort (AUC = 0.797 [95% CI 0.68–0.92]), the validation cohort (AUC = 0.772 [95% CI 0.66–0.88]), and in the combined cohorts (AUC = 0.752 [95% CI 0.67–0.84]). We also established a two-miRNA signature (miR-125b-5p and miR-194-5p) that distinguished PDAC from CCA (validation: AUC = 0.815 [95% CI 0.67–0.96]; and combined cohorts: AUC = 0.814 [95% CI 0.70–0.93]). Our research stands as the largest, multicentric, global profiling study of miRNAs in the bile from patients with pancreaticobiliary disease. We demonstrated their potential as clinically useful diagnostic tools for the detection and differentiation of malignant pancreaticobiliary disease. These bile miRNA biomarkers could be developed to complement current approaches for diagnosing pancreaticobiliary cancers.

**Graphical Abstract:**

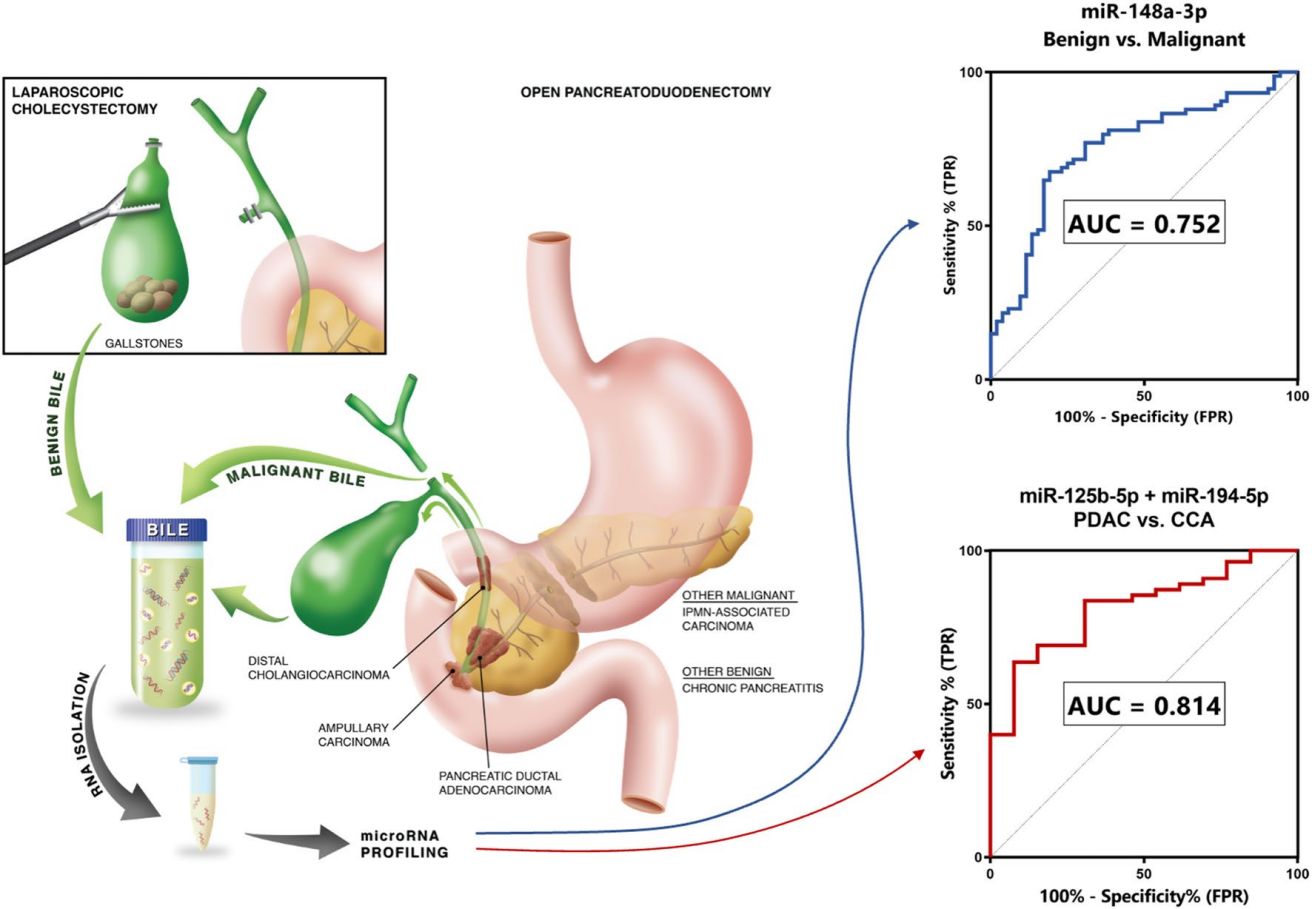

**Supplementary Information:**

The online version contains supplementary material available at 10.1186/s40164-023-00458-3.

## To the editor

Expanding neoadjuvant chemotherapeutic strategies now offer patients distinct treatment options prior to surgery [1, 2]. This major shift in pre-operative care for pancreatic ductal adenocarcinoma (PDAC) has brought to light the longstanding and intricate problem concerning the accurate diagnosis of lesions in the pancreatic head. Patients with malignant (MPD) or benign (BPD) pancreaticobiliary disease often present a diagnostic challenge due to their presentation with similar symptoms, such as obstructive jaundice, as a result of a stricture in the common bile duct (CBD). Moreover, pre-operative diagnostics, including cross-sectional imaging and endoscopic ultrasound-guided fine needle biopsy, can establish a diagnosis in most patients, but some lesions can remain indeterminate [3]. Novel biomarkers are required to improve the diagnostic accuracy, ultimately leading to a decrease in unnecessary major surgeries for benign conditions. The development of reliable biomarkers that can act as adjuncts to standard clinical tests and help diagnose, and distinguish pancreaticobiliary diseases could accelerate treatment decisions and, more importantly, improve survival rates.

Currently, the only biomarker that is used to study recurrence and can support the diagnosis of PDAC is serum CA19-9 [4]. However, benign biliary obstruction with subsequent hyperbilirubinemia can also result in elevated levels of CA19-9. In recent years, microRNAs (miRNAs) have emerged as important players in tumorigenesis in different cancers, including pancreaticobiliary tumors [5]. They are a class of short RNAs that consist of approximately 18–25 non-coding nucleotides. miRNAs regulate gene expression at a post-transcriptional level, affecting protein levels and, as such, form an integral part of many biological processes. One notable feature of miRNAs is their exceptional stability across different bodily fluids like blood and bile, making them well-suited for rapid analysis [5]. Additionally, they show specific expression between tumor types, which is key in cancer biomarker discovery. To date, few studies have demonstrated the presence of miRNAs in bile [6].

Bile is in close proximity, and often in direct contact with pancreaticobiliary tumors and their microenvironment. Interestingly, bile composition is known to be involved in the development of pancreaticobiliary cancers, for example, by reducing susceptibility to apoptosis and promoting cell cycle progression [7]. It can be easily collected preoperatively during endoscopic retrograde cholangiopancreatography (ERCP) or at percutaneous transhepatic biliary drainage (PTBD) procedures for biomarker assessment. Thus, bile-based miRNAs could potentially complement the standard analysis of cytological brushings taken at ERCP or PTBD.

In this study, we aimed to identify bile-based miRNA signatures to discriminate malignant from benign pancreaticobiliary disease by global miRNA profiling of a large, prospective and multicentric cohort of bile samples.

## Prospective biobanking of bile samples from benign and malignant pancreaticobiliary disease

**Fig. 1:**
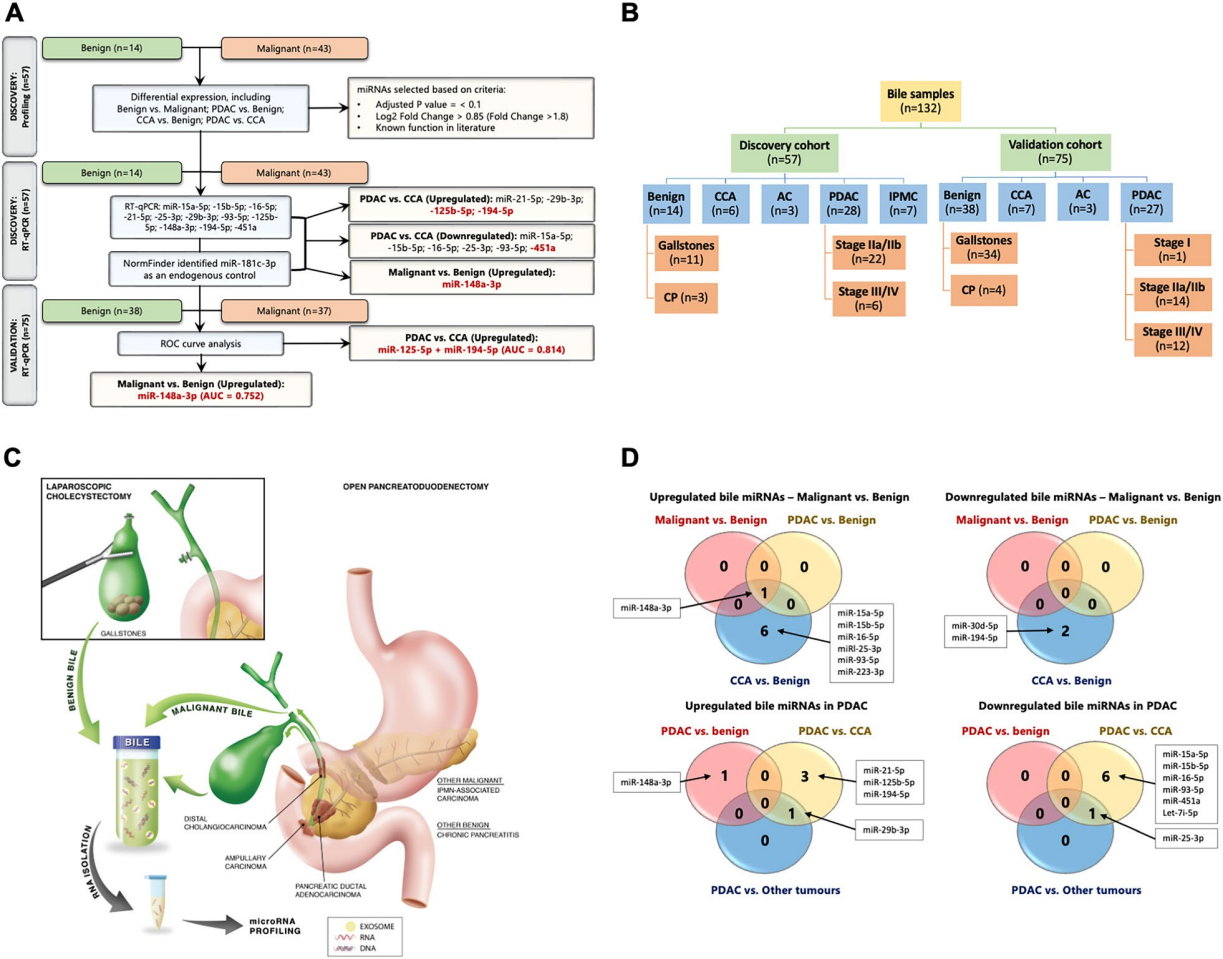
**A** The study design describes 3 phases of our study: discovery, validation and further validation. **B** Bile samples within the patient cohorts specified by disease type (CCA, cholangiocarcinoma; AC, ampullary carcinoma; PDAC, pancreatic ductal adenocarcinoma; IPMC, PDAC originating from intraductal papillary mucinous neoplasm; CP, chronic pancreatitis). **C** Bile samples were collected from laparoscopic cholecystectomy and open pancreatoduodenectomy (PD) for microRNA profiling. **D** Venn diagrams describing differentially expressed microRNAs (miRNAs) in the discovery cohort, when comparing BPD to MPD

The study design is shown in Fig. 1A. The miRNA signatures were established in 3 phases: biomarker discovery, validation of differentially expressed miRNAs, and then further assessment of candidate miRNAs in a second independent cohort. Methods are further described in Additional file 1. Bile samples were collected during laparoscopic cholecystectomy or open pancreatoduodenectomy (PD), and we obtained a discovery (n = 57), and validation cohort (n = 75) (Fig. 1B). These cohorts were clinically well-annotated (Additional file 2: Table S1). The discovery cohort included: cholelithiasis (n = 14), chronic pancreatitis (CP; n = 3), PDAC (stage I/II, n = 22, stage III/IV, n = 5), cholangiocarcinoma (CCA; n = 6), ampullary carcinoma (AC; n = 3) and intraductal papillary mucinous carcinoma (IPMC; n = 7). Most PDAC samples were from stage II disease (n = 22). Mean age was higher in patients with MPD, which corresponds with the late presentation of disease. The validation cohort included: cholelithiasis (n = 34), CP (n = 4), PDAC (n = 27), CCA (n = 7) and AC (n = 3). The number of included samples of PDAC stage I/II (n = 15), was similar to PDAC stage III/IV (n = 12).

## Bile miR-148a-3p is able to discriminate benign from malignant pancreaticobiliary disease

**Fig. 2:**
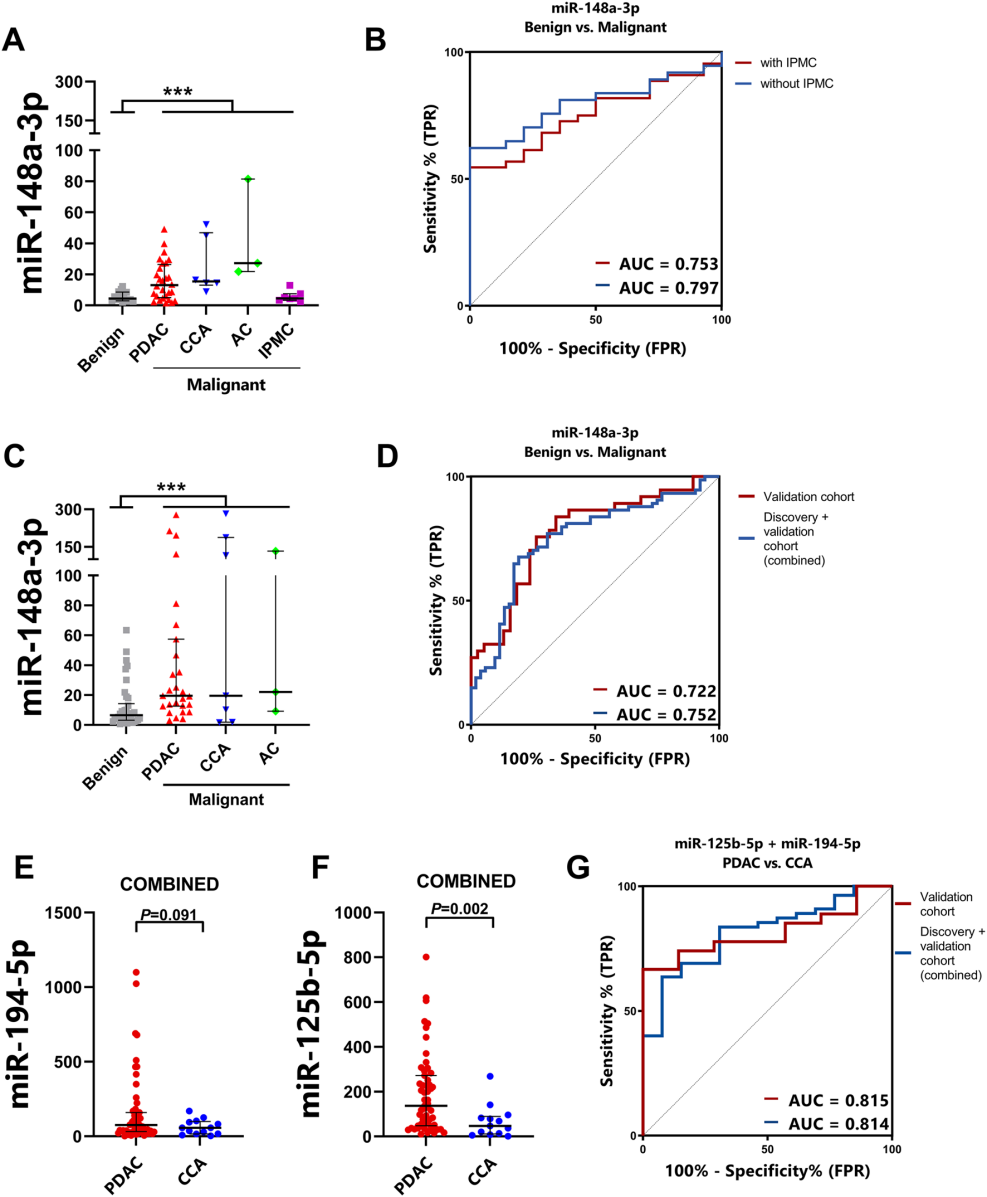
**A** RT-qPCR assessment of miR-148a-3p expression in the discovery cohort when comparing MPD and BPD. **B** A ROC curve was used to demonstrate the diagnostic power of miR-148a-3p in discriminating BPD from MPD including IPCM (AUC = 0.753) and excluding IPMC (AUC = 0.797). **C** Relative expression of miR-148a-3p in the validation cohort showed a significant difference in expression levels, when comparing BPD with MPD. **D** A ROC curve of miR-148a-3p in the validation cohort (AUC = 0.722), as well as when combining the validation cohort with the discovery cohort (AUC = 0.752). **E** Relative expression of miR-194-5p in PDAC vs. CCA in the combined cohort (*P* = 0.002). **F** Relative expression of miR-125b-5p in PDAC vs. CCA (*P* = 0.091) in the combined cohort. **G** Combining miR-125b-5p and miR-194-5p resulted in improved diagnostic power within the validation cohort (AUC = 0.815) and the combined cohort (AUC = 0.814).

Using DEseq2 for the discovery cohort miRNA profiling in BPD (n = 14) and MPD (n = 43), differential miRNA expression analysis revealed several deregulated bile miRNAs (FDR < 0.05) (Fig. 1D). MiR-181c was identified as stable reference gene to normalize future RT-qPCR data. When comparing BPD to MPD, one miRNA (miR-148a-3p) was significantly upregulated in malignant conditions (Fig. 1D). Using RT-qPCR, bile miR-148a-3p was validated (Fig. 2A). Analysis of the ROC curve demonstrated an AUC value of 0.753 (95% CI 0.63–0.88) for miR-148a-3p for differentiating MPD from BPD (Fig. 2B). Notably, miR-148a-3p expression in IPMC resembled BPD more than MPD. Therefore, a second ROC curve with AUC computation was performed excluding IPMC, which led to an AUC of 0.797 (95% CI 0.68–0.92; Fig. 2B) for differentiating MPD from BPD.

## Bile miR-125b-5p and mir-194-5p were significantly upregulated in PDAC compared to CCA

Comparing bile miRNA expression levels between PDAC and CCA (Additional file 3: Fig. S1), we identified 6 downregulated miRNAs (miR-15a5p, miR-15b-5p, miR-16-5p, miR-25-3p, miR-93-5p and miR-451), and 4 upregulated miRNAs (miR-21-5p, miR-29b-5p, miR-125b-5p and miR-194-5p; FDR < 0.05). Even though miR-21-5p did not meet our inclusion criteria, we selected it for RT-qPCR, as it is a well-known oncomiR in PDAC [8]. Bile miR-451a was validated as significantly downregulated (*P* = 0.0353; Additional file 3: Fig. S2) in PDAC compared to CCA. MiR-125b-5p and miR-194-5p were confirmed significantly upregulated in PDAC compared to CCA (Additional file 3: Fig. S3A, B). Bile miR-125b-5p and miR-194-5p demonstrated AUC values of 0.702 (95% CI 0.62–0.96) and 0.786 (95% CI 0.62–0.96) for differentiating PDAC from CCA, respectively (Additional file 3: Fig. S3E, F).

## A validated diagnostic signature for malignant pancreaticobiliary disease

A total of 75 bile samples were obtained for analysis in a second independent cohort (Additional file 2: Table S1). Bile miRNAs that were upregulated (miR-148a-3p, miR-125b-5p and miR-194-5p) in the discovery cohort were selected as candidates for further validation. When comparing MPD (n = 37) with BPD (n = 38), miR-148a-3p demonstrated a statistically significant difference (Fig. 2C), with a diagnostic performance AUC of 0.772 (95% CI 0.67–0.88; Fig. 2D). Also, after combining the discovery and validation cohorts, while excluding IPMC, bile miR-148a-3p showed a robust performance with an AUC value of 0.752 (95% CI 0.67–0.84; Fig. 2D). Next, bile miR-125b-5p and miR-194-5p were validated in PDAC (n = 27) and CCA (n = 7) by RT-qPCR (Fig. 2E, F). When using the combination of miR-125b-5p and miR-194-5p, this revealed AUC values of 0.815 (95% CI 0.67–0.96) and 0.814 (95% CI 0.70–0.93) in the validation cohort and the combined cohort, respectively (Fig. 2G), indicating good discriminatory power for separating PDAC from CCA. For the miRNA signature miR-125b-5p and miR-194-5p, a number of cut-offs with corresponding sensitivity and specificity have been displayed in Additional file 2: Tables S2, S3.

## Discussion

Differential diagnosis of pancreaticobiliary disease can be challenging, and also key to subsequent treatment decisions. With our study, we aimed to help clinical management of pancreaticobiliary diseases by identifying miRNAs that can guide differential diagnosis. MiRNAs are promising clinically-useful biomarkers due to their molecular stability and abundance in different bodily fluids [5]. They can be easily evaluated by means of inexpensive, rapid and widely available RT-qPCR. We found that miR-148a-3p discriminated MPD and BPD with AUC = 0.752, and that miR-125b-5p plus miR-194-5p showed an AUC of 0.814 for distinguishing PDAC from CCA. Interestingly, these miRNAs have been previously associated with PDAC. MiR-148a may play a tumor suppressive role in tumorigenesis, as Liffers et al. discovered that miR-148a directly influences CDC25 expression by binding CDC25B-3′UTR [9]. CDC25 proteins regulate CDK/cyclin complex activation and increased expression can lead to G2/M checkpoint exit and loss of DNA damage repair mechanisms [9]. Furthermore, significant miR-125b upregulation was observed in gemcitabine-resistant cells and associated with a more mesenchymal phenotype [10]. Interestingly, serum miR-194-5p has previously been described as predictive biomarker and was associated with early PDAC progression during FOLFIRINOX treatment [11].

Previous studies suggested that diagnostic performance could be improved by combination with blood-based miRNAs, proteins or serum CA19-9 [12]. The absence of data on CA19-9 might be a potential shortcoming of our study, and we acknowledge that this would be interesting for a subgroup analysis and evaluation of the diagnostic performance compared to bile miRNAs. Moreover, not all candidate miRNAs could be validated using RT-qPCR. Therefore, multi-miRNA classifier analysis was limited to the number of validated miRNAs. However, we feel that our current study overcomes the methodological limitations of many previous studies, by (1) selecting candidate miRNAs after profiling 800 well-annotated miRNAs, (2) performing comprehensive validation of selected miRNAs in a discovery and validation cohort, (3) using profiling data and evidence-based strategies to determine the most robust endogenous normalizer across disease groups. Furthermore, this is the first study to make comparisons between MPD (i.e. PDAC vs. CCA), and to also compare these diseases with BPD.

## Conclusions

One of the major bottlenecks in biomarker discovery is a lack of candidates that are successfully validated. Only through rigorous and standardized testing in diverse representative cohorts can clinically valuable biomarkers be discovered. Our study presents the largest global profiling of miRNAs in the bile from patients with BPD and MPD. We successfully demonstrated the diagnostic potential of bile-based miR-148a-3p, and the two-miRNA panel miR-125b-5p and miR-194-5p as diagnostic tools in MPD (see Additional file 4). Our results support the use of these biomarkers in bile aspirated at ERCP or PTBD procedures to help diagnose patients in combination with standard tests. Ultimately, this approach has the potential to enable clinicians to initiate effective treatment in a timely fashion, and prevent unnecessary interventions, and ultimately leading to improved survival outcomes.

## Supplementary Information

**Additional file 1.** Additional methods.

**Additional file 2: Table S1.** Clinicopathological patient characteristics. **Table S2.** Estimated sensitivity and specificity based on ROC cut-off values of microRNA panel miR-125b-5p and miR-1294-5p in the validation cohort.** Table S3.** Estimated sensitivity and specificity based on ROC cut-off values of microRNA panel miR-125b-5p and miR-1294-5p in the combined cohort.

**Additional file 3: Figure S1.** Differentially expressed miRNAs in cholangiocarcinoma, when comparing pancreaticobiliary diseases in the discovery cohort. **Figure S2.** RT-qPCR validation of miRNAs that were identified as significantly downregulated in PDAC compared to CCA by NanoString nCounter profiling. **Figure S3.** RT-qPCR validation of miRNAs that were identified as significantly upregulated in PDAC compared to CCA by NanoString nCounter profiling.

**Additional file 4.** Summarizing figure/graphical abstract: a summary of our study on bile miRNA biomarker discovery and validation.

## Data Availability

The datasets used and/or analyzed during the current study are available from the corresponding author on reasonable request.

## Abbreviations

AC: Ampullary carcinoma
AUC: Area under the ROC curve
BPD: Benign pancreaticobiliary disease
BTC: Biliary-tract cancer
CBD: Common bile duct
CCA: Cholangiocarcinoma
CI: Confidence interval
CP: Chronic pancreatitis
ERCP: Endoscopic retrograde cholangiopancreatography
GB: Gallbladder
IPMN: Intraductal Papillary Mucinous Neoplasm
IPMC: PDAC associated with IPMN
PD: Pancreatoduodenectomy
PDAC: Pancreatic ductal adenocarcinoma
MPD: Malignant pancreaticobiliary disease
ROC: Receive operator characteristic curve
TCGA: The Cancer Genome Atlas
CI: Confidence interval
