# Supplementary material for: A bile-based microRNA signature for differentiating malignant from benign pancreaticobiliary disease
Source: Exp Hematol Oncol. 2023 Dec 1;12:101. doi: 10.1186/s40164-023-00458-3 (PMC10693033; doi:10.1186/s40164-023-00458-3)
Supplement: Supplementary file 1 — Additional file 1. Additional methods. [file 40164_2023_458_MOESM1_ESM.docx]

Additional file 1

**A bile-based microRNA signature for differentiating malignant from benign pancreaticobiliary disease**

Mireia Mato Prado^1,2,†^, Jisce R. Puik^3,4,†^, Leandro Castellano^1,5^, Elena López-Jiménez^1^, Daniel S.K. Liu^1^, Laura L. Meijer^3,4^, Tessa Y.S. Le Large^3,4^, Eleanor Rees^1^, Niccola Funel^6^, Shivan Sivakumar^7^, Stephen P. Pereira^8^, Geert Kazemier^3,4^, Babs M. Zonderhuis^3,4^, Joris I. Erdmann^3,4^, Rutger-Jan Swijnenburg^3,4^, Andrea Frilling^9^, Long R. Jiao^9^, Justin Stebbing^1,10^, Elisa Giovannetti^4,11,*^, Jonathan Krell^1,‡,*^ & Adam E. Frampton^1,9,12,13,‡,*^

Corresponding authors

Email: [adam.frampton@surrey.ac.uk](mailto:adam.frampton@surrey.ac.uk) or [e.giovannetti@amsterdamumc.nl](mailto:e.giovannetti@amsterdamumc.nl)

**Additional Methods**

**Study design**

The objectives were to identify bile miRNAs able to separate BPD from MPD, and then discriminate PDAC from CCA. The study design is shown in **Fig. 1A**. This study was conducted in 3 phases, including: biomarker identification in a discovery cohort, validation of candidate miRNAs in the same discovery cohort, and further validation in an independent cohort (**Fig. 1B**).

**Sample collection**

Patients aged 18 years and older were prospectively enrolled between 2013 and 2018 at three medical centers: Hammersmith Hospital, Imperial College London NHS Trust (United Kingdom), Amsterdam UMC location VUmc (the Netherlands) and University Hospital of Pisa (Italy). Samples were obtained from patients who were able to give informed consent and in accordance with protocols that are approved by the local ethics committees. Benign bile samples were taken from the gallbladder (GB) after laparoscopic cholecystectomy was performed for uncomplicated gallstones, and from the GB and/or common bile duct (CBD) during pancreatoduodenectomy (PD) for chronic pancreatitis (CP) (**Fig. 1C**). Malignant bile samples were obtained from the GB and/or CBD from patients who underwent tumor resection by PD for a known PDAC, CCA, ampullary carcinoma (AC), or PDAC originating from intraductal papillary mucinous neoplasm (IPMC) (**Fig. 1C**). All patients were chemo-naïve. Bile samples were obtained using a sterile syringe and stored in RNase-free Eppendorf tubes at -80°C for further use.

**RNA isolation**

RNA was isolated from bile specimens using an adapted protocol from TRIzol® LS reagent (Invitrogen) in accordance with the manufacturer’s instructions. Bile samples of 1 mL were thawed on ice and centrifuged at 3,000 x *g* for 10 minutes at 4˚C to remove cellular debris. The supernatant was filtered using a 0.20 µm Minisart® filter (Sartorius) and subsequently lysed in 1 mL of TRIzol® LS reagent in a 2 mL Eppendorf tube. Immediately, 240 µL of chloroform was added to the sample. After shaking it vigorously for 15 seconds, it was centrifuged at 12,000 x *g* for 15 minutes at 4˚C. The aqueous upper phase containing the RNA was transferred to a new 2 mL tube and an equal amount of chloroform was added. It was centrifuged at 12,000 x *g* for 8 minutes at 4˚C. Again, the upper phase was transferred to a new 2 mL tube. Next, 670 µL of isopropanol was added for precipitation purposes. Samples were vortexed for 20 seconds at full speed, after which they were centrifuged at full speed 16,500 x *g* for 30 minutes at 4˚C to precipitate the RNA and form a pellet. The supernatant was discarded and the pellet was washed twice using 70% ethanol. Finally, total RNA, including small RNA, was eluted in 20 µL of RNase free water.

**MicroRNA NanoString nCounter profiling**

To obtain miRNA expression profiles from total RNA, nCounter^®^ Human v3 miRNA Expression Array (NanoString Technologies) was used. This array measures 800 human miRNAs in each sample. nCounter^®^ miRNA sample preparation was performed according to the manufacturer’s instructions. An input of 100 ng purified total RNA was used as recommended. Candidate miRNAs were selected from the discovery experiment based upon fold change > 1.8 and adjusted *p* < 0.1, and known function in the literature.

**Quantitative reverse transcription polymerase chain reaction (RT-qPCR) validation**

Expression levels of miRNAs were validated by RT-qPCR. Using TaqMan microRNA Reverse Transcription Kit (Applied Biosystems, Carlsbad, CA), 10 ng of RNA samples was reverse transcribed. Samples were added onto a 48-well PCR plate (Applied Biosystems) and incubated in a PCR thermal cycler according to the manufacturer’s protocol (Veriti, Applied Biosystems). Specific miRNA TaqMan probes (ThermoFisher Scientific) were used in the reverse transcription including miR15a-5p, miR-15b-5p, miR-16-5p, miR-21-5p, miR-25-3p, miR-29b-3p, miR-93-5p, miR-125b-5p, miR-148a-3p, miR-181c and miR-194-5p and miR-451a.

Quantitative PCR reactions were performed in duplicate using miRNA species TaqMan probes and an endogenous control (miR-181c) following manufacturer’s instructions (ThermoFisher Scientific). The PCR amplification of mature miRNAs consists of repeated thermal cycles involving three sequential steps: 2 minutes at 50°C, then 10 minutes at 95°C, followed by 40 cycles at 95°C for 15 seconds and 60°C for 60 seconds (StepOne Plus, Applied Biosystems). Relative expression levels of each miRNA were calculated using the following equation: 2-ΔCt, where Ct = Cttarget – Ctcontrol, and Ct is the cycle threshold or the number of cycles necessary for the fluorescence signal to cross the threshold in the real-time PCR [1]. MiRNAs with a Ct value > 38 were deemed to be not detected.

**Statistical analysis**

Bile miRNA profiling analyses were performed in R using nCounter^®^ raw values as input for the Bioconductor software package DESeq2 [2]. Bile miRNA expression was normalized to an endogenous control, determined by NormFinder software [3]. Normalized ∆CT were used to calculate relative miRNA expression fold change as 2^-∆∆Ct^ [1]. Differential miRNA expression between groups at RT-qPCR was analyzed using the Mann-Whitney *U*-test (one or two-tailed), in which a *p*-value of *p* < 0.05 was considered statistically significant. ROC curve analysis was conducted for each significantly deregulated and validated miRNA, computing an AUC and 95% confidence intervals (CI). An AUC of 1.0 represents a perfect biomarker, whereas an AUC of 0.5 indicates a result that could be expected by random chance. An AUC of ≥ 0.75 is considered good and an AUC ≥ 0.9 excellent. To establish a miRNA classifier, forced-entry multivariate binary logistic regression analysis was performed. miRNAs were entered in a single step without stepwise selection. Predicted probabilities were calculated and used to generate the ROC curve of the combined miRNA panel. Statistical analyses were performed using SPSS software (version 28, IBM) or GraphPad (version 9.3.1., Prism).

**References**

1. Livak KJ ST. Analysis of relative gene expression data using real-time quantative PCR and the 2(-Delta Delta C(T)) Method. Methods. 2001;25(4):402-8.

2. Love MI, Huber WA, S. Moderated estimation of fold change and dispersion for RNA-seq data with DESeq2. Genome Biology. 2014;15:550.

3. Andersen CL, Jensen JL, Ørntoft TF. Normalization of Real-Time Quantitative Reverse Transcription-PCR Data: A Model-Based Variance Estimation Approach to Identify Genes Suited for Normalization, Applied to Bladder and Colon Cancer Data Sets. Cancer Res. 2004;64:5245 - 50.
