# Supplementary material for: A bile-based microRNA signature for differentiating malignant from benign pancreaticobiliary disease
Source: Exp Hematol Oncol. 2023 Dec 1;12:101. doi: 10.1186/s40164-023-00458-3 (PMC10693033; doi:10.1186/s40164-023-00458-3)
Supplement: Supplementary file 2 — Additional file 2: Table S1. Clinicopathological patient characteristics. Table S2. Estimated sensitivity and specificity based on ROC cut-off values of microRNA panel miR-125b-5p and miR-1294-5p in the validation cohort. Table S3. Estimated sensitivity and specificity based on ROC cut-off values of microRNA panel miR-125b-5p and miR-1294-5p in the combined cohort. [file 40164_2023_458_MOESM2_ESM.docx]

Additional file 2

**A bile-based microRNA signature for differentiating malignant from benign pancreaticobiliary disease**

Mireia Mato Prado^1,2,†^, Jisce R. Puik^3,4,†^, Leandro Castellano^1,5^, Elena López-Jiménez^1^, Daniel S.K. Liu^1^, Laura L. Meijer^3,4^, Tessa Y.S. Le Large^3,4^, Eleanor Rees^1^, Niccola Funel^6^, Shivan Sivakumar^7^, Stephen P. Pereira^8^, Geert Kazemier^3,4^, Babs M. Zonderhuis^3,4^, Joris I. Erdmann^3,4^, Rutger-Jan Swijnenburg^3,4^, Andrea Frilling^9^, Long R. Jiao^9^, Justin Stebbing^1,10^, Elisa Giovannetti^4,11,*^, Jonathan Krell^1,‡,*^ & Adam E. Frampton^1,9,12,13,‡,*^

Corresponding authors

Email: [adam.frampton@surrey.ac.uk](mailto:adam.frampton@surrey.ac.uk) or [e.giovannetti@amsterdamumc.nl](mailto:e.giovannetti@amsterdamumc.nl)

|  | **Discovery cohort (n = 57)** | | | | | **Validation cohort (n = 75)** | | | |
| --- | --- | --- | --- | --- | --- | --- | --- | --- | --- |
|  | **Benign disease** (n = 14) | **PDAC** (n = 28) | **CCA** (n = 6) | **AC** (n = 3) | **IPMC** (n = 6) | **Benign disease** (n = 38) | **PDAC** (n = 27) | **CCA** (n = 7) | **AC** (n = 3) |
| **Age – years** Mean (± SD) | 50 (± 12) | 64 (± 7) | 67 (± 15) | 70 (± 8) | 65 (± 6) | 53 (± 13) | 64 (± 9) | 66 (± 9) | 62 (± 8) |
| **Sex – no. (%)** |  | | | | |  |  |  |  |
| Male | 7 (47) | 17 (61) | 4 (67) | 3 (100) | 3 (50) | 16 (42) | 16 (59) | 4 (57) | 3 (100) |
| Female | 8 (53) | 11 (39) | 2 (33) | 0 (0) | 3 (50) | 21 (55) | 11 (41) | 3 (43) | 0 (0) |
| **Stage^+,^*– no. (%)** |  | | | | |  |  |  |  |
| I | - | 0 (0) | 1 (17) | 1 (33) | 0 (0) | - | 1 (4) | 0 (0) | 0 (0) |
| II | - | 22 (79) | 2 (33) | 2 (66) | 0 (0) | - | 14 (52) | 4 (57) | 3 (100) |
| III | - | 2 (7) | 2 (33) | 0 (0) | 6 (100) | - | 8 (30) | 1 (14) | 0 (0) |
| IV | - | 4 (14) | 1 (17) | 0 (0) | 0 (0) | - | 4 (14) | 0 (0) | 0 (0) |

**Table S1.** Clinicopathological patient characteristics.

Abbreviations: PDAC: pancreatic ductal adenocarcinoma; CCA: cholangiocarcinoma; AC: ampullary cancer; IPMC: PDAC originating from intraductal papillary mucinous neoplasm. ^+^In accordance with the eight edition of the AJCC Cancer Staging System. *****All patients were WHO score 0 to 1.

**Table S2.** Estimated sensitivity and specificity based on ROC cut-off values of microRNA panel miR-125b-5p and miR-1294-5p in the validation cohort. Various cut-off levels were used to overcome bias in sensitivity and specificity caused by data-driven selection of optimal cutoff values [1].

| Cut-off |  | Sensitivity% | 95% Confidence Interval | Specificity% | 95% Confidence Interval |
| --- | --- | --- | --- | --- | --- |
| > 0.4710 |  | 100 | 88% to 100% | 14 | 1% to 51% |
| > 0.5355 |  | 89 | 72% to 96% | 29 | 5% to 64% |
| > 0.5642 |  | 85 | 68% to 94% | 43 | 16% to 75% |
| > 0.7060 |  | 74 | 55% to 87% | 86 | 49% to 99% |
| > 0.8632 |  | 67 | 48% to 81% | 100 | 65% to 100% |

**Table S3.** Estimated sensitivity and specificity based on ROC cut-off values of microRNA panel miR-125b-5p and miR-1294-5p in the combined cohort. Various cut-off levels were used to overcome bias in sensitivity and specificity caused by data-driven selection of optimal cutoff values [1].

| Cut-off | Sensitivity% | 95% Confidence Interval | Specificity% | 95% Confidence Interval |
| --- | --- | --- | --- | --- |
| > 0.4758 | 100 | 94% to 100% | 15 | 2% to 45% |
| > 0.5650 | 91 | 80% to 97% | 31 | 9% to 61% |
| > 0.6106 | 87 | 76% to 95% | 46 | 19% to 75% |
| > 0.6633 | 84 | 71% to 92% | 69 | 39% to 91% |
| > 0.8000 | 69 | 55% to 81% | 85 | 55% to 98% |
| > 0.9487 | 40 | 27% to 54% | 100 | 75% to 100% |

**Additional Reference**

1. Leeflang MM, Moons KG, Reitsma JB, Zwinderman AH. Bias in sensitivity and specificity caused by data-driven selection of optimal cutoff values: mechanisms, magnitude, and solutions. Clin Chem. 2008;54(4):729-37.
