# Supplementary material for: A bile-based microRNA signature for differentiating malignant from benign pancreaticobiliary disease
Source: Exp Hematol Oncol. 2023 Dec 1;12:101. doi: 10.1186/s40164-023-00458-3 (PMC10693033; doi:10.1186/s40164-023-00458-3)
Supplement: Supplementary file 3 — Additional file 3: Figure S1. Differentially expressed miRNAs in cholangiocarcinoma, when comparing pancreaticobiliary diseases in the discovery cohort. Figure S2. RT-qPCR validation of miRNAs that were identified as significantly downregulated in PDAC compared to CCA by NanoString nCounter profiling. Figure S3. RT-qPCR validation of miRNAs that were identified as significantly upregulated in PDAC compared to CCA by NanoString nCounter profiling. [file 40164_2023_458_MOESM3_ESM.docx]

Additional file 3

**A bile-based microRNA signature for differentiating malignant from benign pancreaticobiliary disease**

Mireia Mato Prado^1,2,†^, Jisce R. Puik^3,4,†^, Leandro Castellano^1,5^, Elena López-Jiménez^1^, Daniel S.K. Liu^1^, Laura L. Meijer^3,4^, Tessa Y.S. Le Large^3,4^, Eleanor Rees^1^, Niccola Funel^6^, Shivan Sivakumar^7^, Stephen P. Pereira^8^, Geert Kazemier^3,4^, Babs M. Zonderhuis^3,4^, Joris I. Erdmann^3,4^, Rutger-Jan Swijnenburg^3,4^, Andrea Frilling^9^, Long R. Jiao^9^, Justin Stebbing^1,10^, Elisa Giovannetti^4,11,*^, Jonathan Krell^1,‡,*^ & Adam E. Frampton^1,9,12,13,‡,*^

Corresponding authors

Email: [adam.frampton@surrey.ac.uk](mailto:adam.frampton@surrey.ac.uk) or [e.giovannetti@amsterdamumc.nl](mailto:e.giovannetti@amsterdamumc.nl)


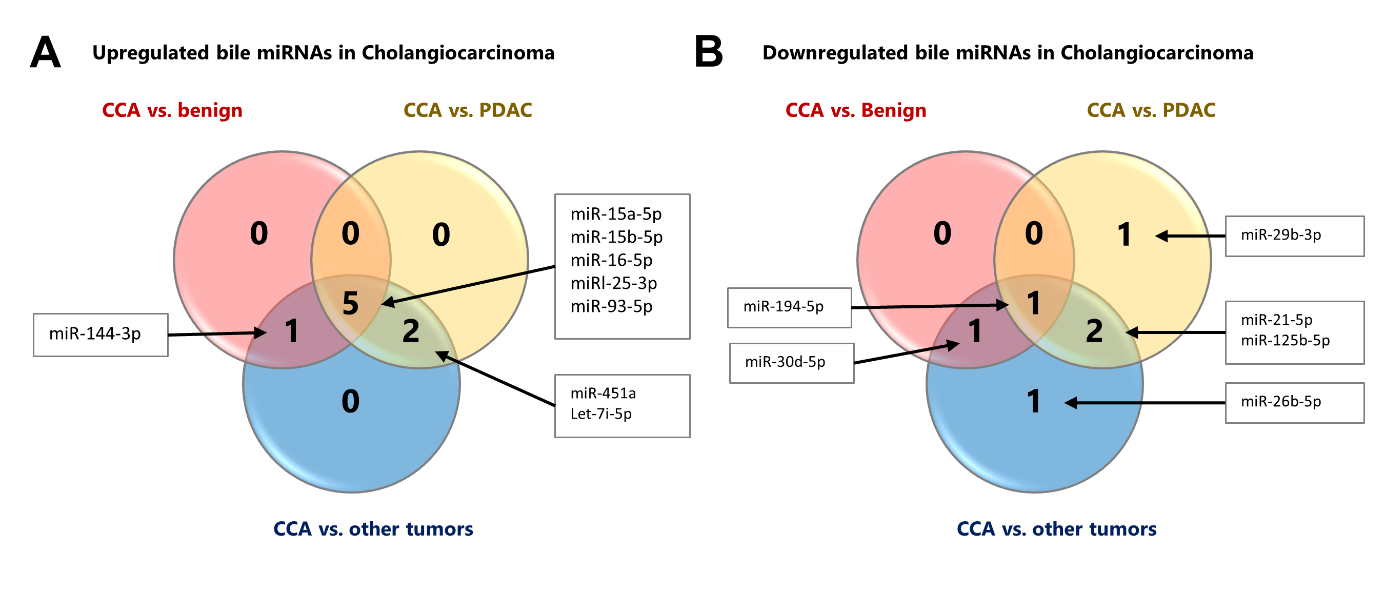


**Figure S1:** Differentially expressed miRNAs in cholangiocarcinoma, when comparing pancreaticobiliary diseases in the discovery cohort. (A) Venn diagrams show upregulated miRNAs and (B) downregulated miRNAs in cholangiocarcinoma.

**
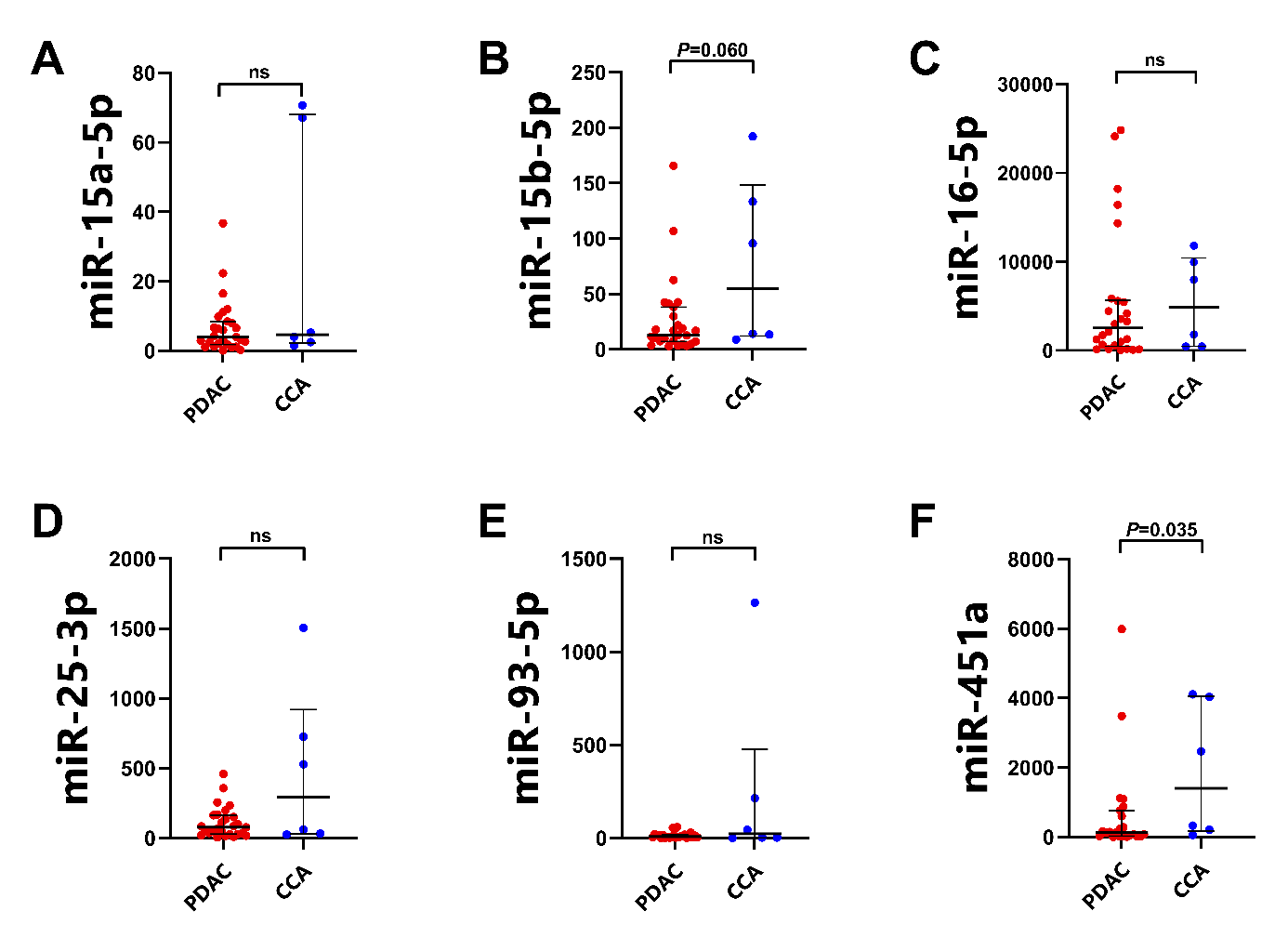
 Figure S2:** RT-qPCR validation of miRNAs that were identified as significantly downregulated in PDAC compared to CCA by NanoString nCounter profiling. Relative expression levels of (A) miR-15a-5p, (B) miR-15b-5p, (C) miR-16-5p, (D) miR-25-3p, (E) miR-93-5p, (F) miR-451a.


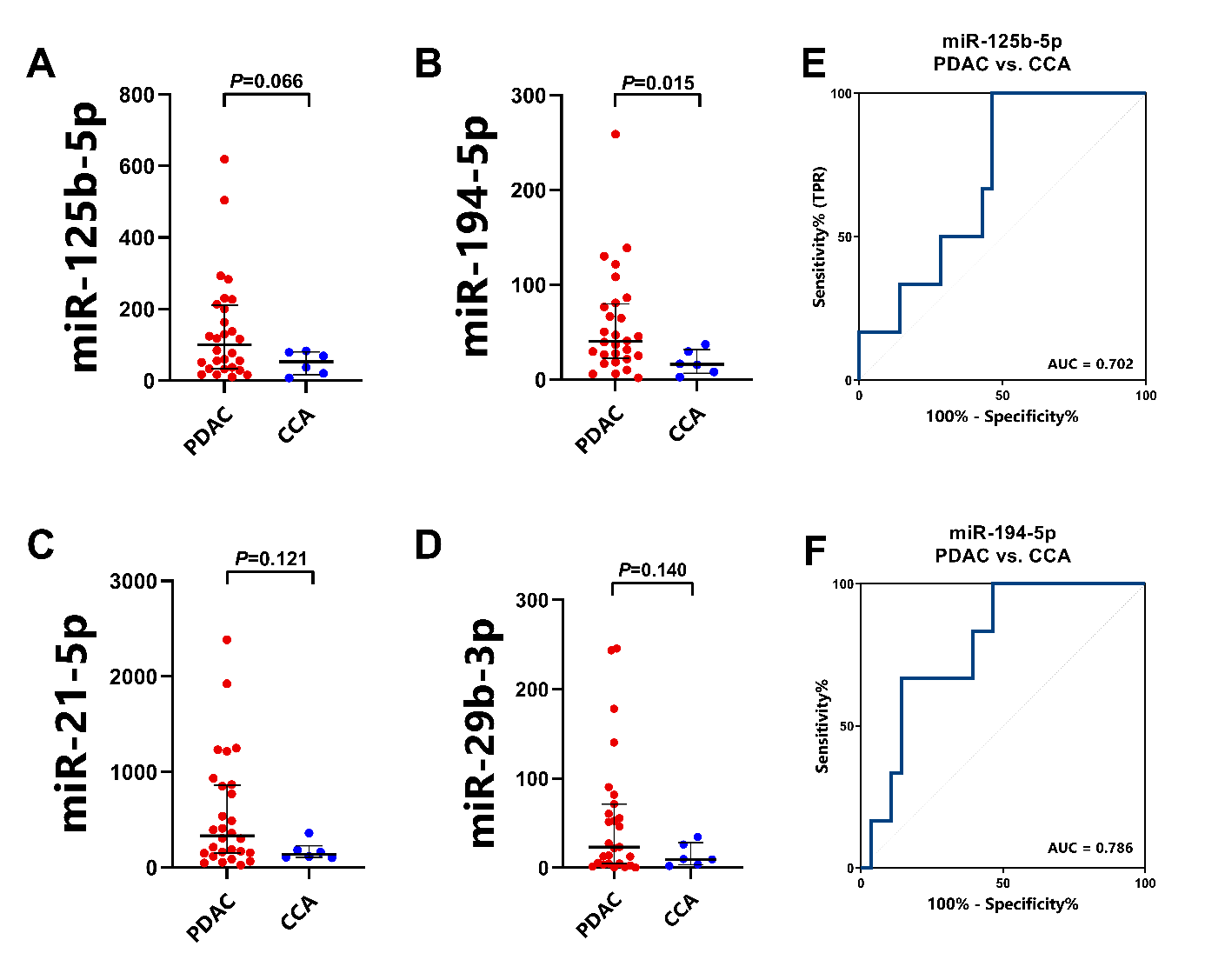


**Figure S3:** RT-qPCR validation of miRNAs that were identified as significantly upregulated in PDAC compared to CCA by NanoString nCounter profiling. Relative expression levels of (A) miR-21-5p, (B) miR-29b-3p (C) miR-125b-5p, (D) miR-194-5p. (E) ROC curves were used to compute AUC values for miR-135b-5p (AUC = 0.702) (F) and for miR-194-5p (AUC = 0.786).
