# Supplementary material for: A bile-based microRNA signature for differentiating malignant from benign pancreaticobiliary disease
Source: Exp Hematol Oncol. 2023 Dec 1;12:101. doi: 10.1186/s40164-023-00458-3 (PMC10693033; doi:10.1186/s40164-023-00458-3)
Supplement: Supplementary file 4 — Additional file 4. Summarizing figure/graphical abstract: a summary of our study on bile miRNA biomarker discovery and validation. [file 40164_2023_458_MOESM4_ESM.docx]

Additional file 4

**A bile-based microRNA signature for differentiating malignant from benign pancreaticobiliary disease**

Mireia Mato Prado^1,2,†^, Jisce R. Puik^3,4,†^, Leandro Castellano^1,5^, Elena López-Jiménez^1^, Daniel S.K. Liu^1^, Laura L. Meijer^3,4^, Tessa Y.S. Le Large^3,4^, Eleanor Rees^1^, Niccola Funel^7^, Shivan Sivakumar^7^, Stephen P. Pereira^8^, Geert Kazemier^3,4^, Babs M. Zonderhuis^3,4^, Joris I. Erdmann^3,4^, Rutger-Jan Swijnenburg^3,4^, Andrea Frilling^9^, Long R. Jiao^9^, Justin Stebbing^1,10^, Elisa Giovannetti^4,11,*^, Jonathan Krell^1,‡,*^ & Adam E. Frampton^1,9,12,13,‡,*^

Corresponding authors

Email: [adam.frampton@surrey.ac.uk](mailto:adam.frampton@surrey.ac.uk) or [e.giovannetti@amsterdamumc.nl](mailto:e.giovannetti@amsterdamumc.nl)


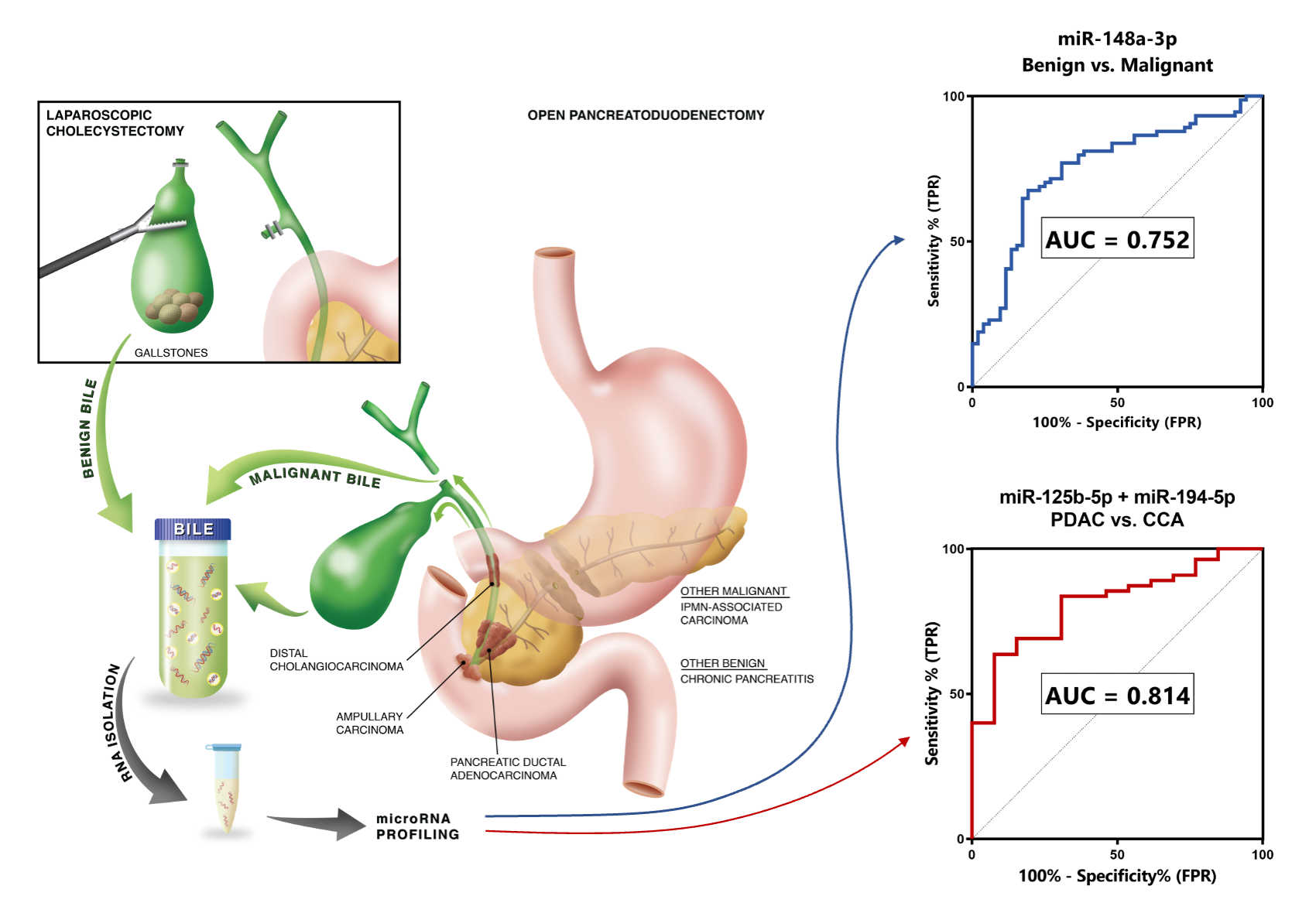


**Summarizing Figure/Graphical Abstract:** A summary of our study on bile miRNA biomarker discovery and validation. Bile miRNA biomarker discovery was performed by global profiling of bile from malignant and benign pancreaticobiliary disease. To establish reliable diagnostic miRNAs, miRNAs were validated using RT-qPCR and then further assessed in a second independent validation cohort. Bile miR-148a-3p discriminated malignant from benign disease with an AUC value of 0.752 [95%CI:0.67-0.84]. We also identified a two-miRNA signature (miR-125b-5p and miR-194-5p) to distinguish pancreatic ductal adenocarcinoma from cholangiocarcinoma with a diagnostic performance AUC of 0.814 [95%CI:0.70-0.93]).
